# The Effect of Vitamin D3 Supplementation on Vitamin D Status and Associated Health Outcomes in Children: A Randomized Controlled Trial

**DOI:** 10.1016/j.tjnut.2026.101493

**Published:** 2026-03-24

**Authors:** Emily Royle, Dominique U Glatt, Emeir M McSorley, Kirsty Pourshahidi, David J Armstrong, Laura Grimley, Mary M Slevin, Cealan O Henry, James E McMullan, R Revuelta Iniesta, Jane T McCluskey, Nigel Gleeson, Pamela J Magee

**Affiliations:** 1 Nutrition Innovation Centre for Food and Health (NICHE), School of Biomedical Sciences, Ulster University, Coleraine, Northern Ireland, United Kingdom; 2 Department of Rheumatology, Altnagelvin Area Hospital, Western Health and Social Care Trust, Londonderry, Northern Ireland, United Kingdom; 3 Public Health and Sports Sciences, Faculty of Health and Life Sciences, Medical School, University of Exeter, Exeter, United Kingdom; 4 Department of Dietetics and Nutrition, Queen Margaret University, Edinburgh, United Kingdom

**Keywords:** vitamin D supplementation, 25(OH)D status, muscle strength, children, balance, immune function, cognitive function, bone turnover markers

## Abstract

**Background:**

Vitamin D is a key regulator of musculoskeletal growth, immune regulation, and cognitive function in children. As children in the United Kingdom (UK) and Ireland are at risk of vitamin D deficiency due to the northern latitude, supplementation is recommended. To our knowledge, the effect of supplementation on status and related health outcomes has not been investigated in children residing in the UK/Ireland.

**Objectives:**

To examine the effect of vitamin D_3_ supplementation on vitamin D status and related health outcomes in children.

**Methods:**

The D vitamin in children study was a double-blind, randomized, placebo-controlled trial, conducted among healthy children (aged 4–11 y). Children received 12 wk of either 10 *μ*g/d vitamin D_3_ or a placebo devoid of vitamin D (control) in the form of an oral spray. The primary outcome, plasma 25-hydroxyvitamin D, and secondary outcomes, including grip strength, balance, cognitive function, winter status, immune markers, and bone turnover markers, were assessed pre- and postintervention.

**Results:**

One hundred eighteen participants completed the study (mean age 8.1 ± 1.8 y; 51% girls). Supplementation significantly increased 25-hydroxyvitamin D concentration (from 66.31 ± 17.26 nmol/L to 69.04 ± 16.93 nmol/L) compared to a decline in the placebo group (63.67 ± 19.48 nmol/L to 56.29 ± 18.58 nmol/L; *P* < 0.001) and prevented deficiency during the extended winter months. No effects were observed on muscle function, cognitive function, immune function, or bone turnover markers.

**Conclusions:**

Vitamin D insufficiency is prevalent in UK/Irish children, and supplementation in the form of an oral spray is effective in achieving/maintaining an adequate status in most children. Further research is warranted to elucidate mechanisms underpinning nonresponse to supplementation and to further investigate the potential beneficial effects of supplementation on cognitive function.

This trial was registered at clinicaltrials.gov as NCT05018988.

## Introduction

Vitamin D deficiency during childhood is associated with severe adverse skeletal health effects leading to an increased risk of developing rickets [1]. The incidence of rickets in the United Kingdom (UK) is 0.48/100,000 in children under 16 y presenting to secondary care. Although evidence for an increasing incidence is limited, rickets persists in UK children, underscoring the importance of preventative strategies [2]. Nutritional rickets represents the severe end of the vitamin D deficiency spectrum [3], but milder forms of deficiency (25‒50 nmol/L) are more common and have been associated with adverse outcomes, including impaired bone mineralization, increased bone turnover [4,5], and reduced muscle strength in adolescents [6,7]. Beyond its role in calcium homeostasis and skeletal development, optimal vitamin D may also influence immune health and cognitive function in infants and adolescents [[8], [9], [10], [11]].

Although global definitions of vitamin D status vary, in the UK, vitamin D deficiency is defined as a circulating 25-hydroxyvitamin D [25(OH)D] concentration of <25 nmol/L [12]. Concentrations of 25‒50 nmol/L are considered insufficient, and >50 nmol/L are considered sufficient [13]. At higher latitudes, vitamin D status is strongly influenced by season due to negligible Ultraviolet B (UVB) radiation exposure throughout the extended winter (November to March) [14]. When cutaneous synthesis is impeded, adequate vitamin D intakes must be met through diet and/or supplementation [15], yet consistently low dietary vitamin D intakes are reported in UK and Irish children [[16], [17], [18]]. In the most recent National Diet and Nutrition Survey in the UK, 10% of children aged 4–10 y had low vitamin D status [18]. Notably, within the National Diet and Nutrition Survey dataset, only 6 children aged 4‒10 y were sampled for 25(OH)D concentrations in Northern Ireland, despite a relatively narrow latitude range (55‒56°N), which provides a distinct context for examining vitamin D status given its mixed urban-rural characteristics. Previous research in children and adolescents at similar (51‒55°N) or higher latitudes (56‒63°N) has demonstrated that vitamin D supplementation can improve vitamin D status and reduce the risk of deficiency, including studies from the UK [19], Sweden [20], and Denmark [21]. Most studies, however, have been limited to the winter months and narrow age ranges, restricting generalizability. In 2016, UK Government vitamin D recommendations were revised to include children [12], yet no comprehensive trial has evaluated whether supplementation is effective at maintaining or improving 25(OH)D concentrations across children aged 4‒11 y within the UK. Current guidelines of 10 *μ*g/d are based on supporting bone health and evidence for optimal vitamin D intakes to support other health outcomes, such as muscle function, which remains limited, especially in children.

The paucity of data investigating vitamin D status and the effects of supplementation in healthy children shows that further research is warranted. Therefore, the primary aim of this study was to investigate the effect of 10 *μ*g/d vitamin D_3_ supplementation over 12 wk on 25(OH)D concentrations in healthy children aged 4‒11 y. Secondary aims investigated the effect of vitamin D_3_ supplementation on musculoskeletal function, sensorimotor function, and cognitive function, with subset analysis conducted in extended winter supplementation, immune markers, and bone turnover markers. By addressing these gaps, this study aims to provide novel evidence to inform child health policy in the UK, where supplementation is low, fortification is voluntary, and socioeconomic inequalities may exacerbate the risk of deficiency.

## Methods

### Study design

The D vitamin in children study (phase 2) was conducted at the Nutrition Innovation Centre for Food and Health, Ulster University, Coleraine, Northern Ireland. Initial study recruitment and sampling commenced between November 2019 and March 2020, after which the study was postponed owing to the COVID-19 pandemic. Following the recommencement of research, recruitment was completed between August 2021 and August 2023. Eligible children aged 4‒11 y (*n* = 118) were randomly assigned to receive a spray containing either 10 *μ*g/d vitamin D_3_ or a placebo (containing 0 *μ*g vitamin D_3_) matched in taste, smell, appearance, and size of container. Children were randomly allocated in a 1:1 ratio stratified by age categories 4‒6 y, 7‒9 y, and 10‒11 y old. Children from the same family were given the same allocation. All aspects of randomization were carried out independently by the Human Intervention Studies Unit Clinical Trials Manager at Ulster University. Participants were invited to attend 2 90-min appointments, at the beginning and end of the trial. Participants who had originally undertaken the study but were unable to complete the trial due to COVID-19 restrictions were invited to take part when the study recommenced in 2021. Ethical approval was granted from Ulster University’s Research Ethics Committee (REC/19/0069; clinical trials registration: NCT05018988).

### Participants

Recruitment occurred via social media, internal mailing lists, in-person educational health talks, and school parent evenings. Parents contacted the researcher via email or phone, and either the researcher completed the screening questionnaire with the parent via phone or the parent completed an online screening questionnaire via a Quick Response (QR) link. Healthy participants free from chronic disease were invited to either enroll in the trial if not taking a vitamin D supplement, or were willing to abstain from starting vitamin D supplements. Children were excluded from participating if they were prescribed long-term medication that affected vitamin D metabolism; and/or diagnosed with a long-term or exacerbated health condition or disease (children with minor or mild health conditions were not excluded), or using sun beds for the duration of the intervention. All parents and children provided written consent and assent, respectively, at the time of recruitment. Baseline appointments were held at either the Human Intervention Studies Unit or a suitable off-site location, with postintervention appointments for eligible participants held 12 wk later.

### Anthropometry

Weight was measured using electronic SECA α flat scales (SECA Ltd), and height (centimeters) was measured using a SECA 213 portable stadiometer (SECA Ltd). All children had measurements taken in triplicate, wearing light clothing with items removed from pockets where necessary, and without shoes. BMI (in kg/m^2^) was calculated, and the WHO AnthroPlus software for children and adolescents aged 5‒19 y was used to convert into BMI *z*-scores [22].

### Musculoskeletal

Grip strength was assessed by a dynamometer with the participant standing, shoulder adducted, and elbow parallel to the thigh, to assess muscle strength [TKK 5001 Grip-A analog dynamometer (64080136); Takei Scientific Instruments Co. Ltd]. Three measurements were performed with short rests of ≤30 s between each test. An age-specific adapted grip strength of both dominant and nondominant hands was calculated as mean grip strength (kilogram)/BMI [23]. Sensorimotor testing of participants included balance using single-leg stance and tandem stance with a method previously described by Condon and Cremin [24].

### Cognitive function assessment

A battery of cognitive tests was conducted using the well-validated Cambridge Neuropsychological test automated battery (CANTABeclipse, Research Suite; Cambridge Cognition) [25]. Neurocognitive tests were designed to assess a range of cognitive domains at pre- and postintervention through nonverbal stimuli and touch screen methodology. The following tests were performed: spatial span (SSP), simple reaction time (s-RTI), 5-choice reaction time (5-RTI), and rapid visual information processing (RVP). Full details of each test procedure are reported elsewhere [26,27]. Briefly, all participants were asked to complete a motor screening test to familiarize themselves with the battery. Following this, the SSP task measured the capacity of immediate memory through the ability to recall the order of color change of boxes in a series by pressing the highlighted boxes in the correct recall sequence. The dependent measure was the longest sequence recalled correctly. Attention was assessed by both s-RTI and 5-RTI; participants were required to react to a yellow dot appearing inside either 1 (s-RTI) or 5 (5-RTI) circles in the center of the computer screen. Participants were instructed to release the press pad and touch the dot as quickly as possible. The dependent variable was the mean latency measured in milliseconds between the dot’s appearance and the release of the press pad. The RVP is designed to assess sustained attention capacity through a 4-min visual continuous performance task. Briefly, digits ranging from 2 to 9 are presented 1 at a time in a random order on the screen. Participants were required to respond when the target sequence was presented; the target sequence was modified for either age 4‒6 y or 7‒11 y. The procedure for assessing RVP is described in full detail elsewhere [28].

### Questionnaire measures

All questionnaires were self-reported except for the validated food frequency questionnaire [29], which was administered by the research team. Mean daily vitamin D intakes were calculated for each participant, based on dietary sources only, and did not include vitamin D from the supplement given during the study. Parents provided information on child and parent demographics and lifestyle factors. The validated children’s physical activity questionnaire was also completed at baseline [30,31]. At the recommencement of the study, a COVID-19 lockdown questionnaire was introduced to document any known changes in vitamin D supplementation, vitamin D-rich food intakes, and outdoor activity, which are all associated with 25(OH)D concentrations. To determine skin phototype, an adapted Fitzpatrick scale was utilized to determine skin pigmentation type [32].

### Intervention

Both the supplement and the inactive placebo were provided in kind by BetterYou Ltd (BetterYou) in oral spray form, 10 mL. Each active vitamin D_3_ spray consisted of xylitol, water, acacia gum, cholecalciferol (vitamin D_3_), sunflower lecithin, citric acid, preservative: potassium sorbate, and peppermint oil. An inactive placebo spray identical but lacking cholecalciferol (vitamin D_3_) served as the control. All sprays were packaged unlabeled with no distinguishing markings and were delivered to the clinical trials manager and distributed to the research team to ensure double blinding. Participants were provided with standardized verbal and written instructions on spray use, including priming the spray before use and a physical demonstration by the researcher with a placebo spray. Dose standardization was achieved by instructing parents to administer 1 spray per day, corresponding with the manufacturer-labeled dose of 10 *μ*g per vitamin D_3_ spray. The oral spray was administered daily either in the morning or evening to the mucosal buccal membrane, either by the child or assisted by the parent, depending on the child’s ability. Participants were requested to return the spray bottles at the end of the study. Compliance was assessed through weighing the spray bottles pre- and postuse, as well as through a star chart completed by participants.

### Blood collection and laboratory analysis

Pre- and postintervention, a nonfasting 20 mL venous blood sample was obtained from the antecubital fossa by a trained pediatric phlebotomist. Sample preparation and fractionation were undertaken within 4 h of sampling, and blood aliquots were stored at ‒80°C until batch analysis. EDTA 4 mL samples were sent to the Clinical Biochemistry Laboratory, Altnagelvin Area Hospital, for intact parathyroid hormone (iPTH) and analyzed using a Cobas 800 clinical biochemistry analyser (e602 immunoassay module; Roche Diagnostics Ltd) (Coefficient of Variation (CV) 1.7).

### Plasma 25-(OH)D quantification

Plasma 25(OH)D concentrations were analyzed by the gold standard liquid chromatography tandem mass spectrometry (LC–MS/MS). A reverse phase [phase A consisted of 50:50 (*vol/vol*) water: acetonitrile containing 0.1% formic acid as a modifier; phase B consisted of acetonitrile with 0.1% formic acid] chromatography [Polar C18 2.6 μm (3.0 × 100 mm) column] (Phenomenex). Mass spectrometry was performed using an API 4000 mass spectrometer (AB SCIEX) by means of turbo spray ionization in positive ion mode. The multiple reaction monitoring transition for 25(OH)D_3_ was used as an acquisition method. Quantification was performed by internal standard ratios using a linear regression model. Vitamin D status cut-off thresholds for 25(OH)D concentrations follow UK convention, where <25 nmol/L is deficient, 25‒50 nmol/L is insufficient, and >50 nmol/L is sufficient [12,13].

### Blood biomarker analysis

To determine the effect of vitamin D supplementation on immune markers and bone turnover markers, a subset of participants was selected based on baseline vitamin D status (*n* = 54). Participants were categorized into either insufficient (≤50 nmol/L) or sufficient (≥50 nmol/L) vitamin D status and were matched according to sex, age, and BMI within the vitamin D or placebo group. Inflammatory markers including C-reactive protein, interferon γ, TNF-α and IL-1β, IL-2, IL-4, IL-6, IL-8, IL-10, IL-12p70, and IL13 were selected to explore changes in innate and adaptive cytokines that may be modulated by vitamin D. The inflammatory markers panel was analyzed via electrochemiluminescence-based Meso Scale Discovery immunoassay following manufacturer’s instructions (Meso Scale Discovery V-Plex Multi-Spot Assay System, K151A9H-1, and S-Plex Multi-Spot Assay System, K151AKV-1 and K151Y9S-1, Meso Scale Discovery, Inc. Rockville, USA.). Concentrations were expressed as mean ± SD in fg/mL. The lower limits of detection (LLOD) (pg/mL) for C-reactive protein and IL-8 were 4.96 and 0.07, respectively. The LLOD (fg/mL) for interferon γ, TNF-α, IL-1β, IL-2, IL-4, IL-6, IL-10, IL-12p70, and IL-13 were 10.8, 19.8, 180.0, 23.3, 33.8, 36.4, 44.8, 187.0, and 8.20, respectively. Sample concentrations below the LLOD were input as LLOD. (Timepoint 1 CVs: 11.3%, 8.7%, 28.6%, 10.8%, 6.9%, 4.5%, 7.1%, 4.7%, and 12.1%; timepoint 2 CVs: 4.6%, 4.8%, 15.3%, 5.2%, 7.3%, 3.5%, 5.3%, 5.3%, and 7.5% respectively). Bone turnover markers were assessed via immunoassay: C-terminal telopeptide of type 1 collagen (CTX) and procollagen type 1 intact N-terminal propeptide (P1NP), (Roche, F. Hoffmann-La Roche Ltd). CVs: 28.1% and 22.2% at timepoint 1 and 34.9% and 24.9% timepoint 2, respectively (Belfast City Hospital, Belfast, Northern Ireland, December 2023).

### Statistical analyses

The sample size was estimated using data from Mortensen et al. [20]. In that study in children, a daily dose of 10 *μ*g/d vitamin D_3_ resulted in a mean change in serum 25(OH)D concentration of 4.9 nmol/L, with a SD of 8 nmol/L. Assuming a 2-sided significance level of 0.05 and 90% power, a minimum of 47 participants per group was required to detect this difference. To account for an anticipated 20% dropout rate, an additional 12 participants were recruited per group, giving a total required sample size of 118. All secondary outcomes were exploratory, and the study was not powered to detect differences in these outcomes. All analysis was conducted through the Statistical Package for the Social Sciences; significance was set at *P* < 0.05 (IBM SPSS Statistics for Windows, version 29.0, IBM Corp.). Following the Consolidated Standards of Reporting Trials guidelines, analyses were completed using intention-to-treat analysis. All data was double entered, and data that were missed due to child refusal or not collected at time point 2 were imputed by last observation carried forward before analysis. Normality was tested using visual inspection of histograms and the Kolmogorov-Smirnov normality test. Descriptive statistics were performed for baseline characteristics, with differences between groups compared using a χ^2^ test for categorical data and an independent t-test for continuous data.

The primary outcome was analyzed using analysis of covariance (ANCOVA) with postintervention concentration as the dependent variable, adjusting for baseline 25(OH)D concentration, age, BMI, dietary vitamin D intake, physical activity (sedentary minutes), season, and skin pigmentation type. Children from the same family were included in the trial, and the overall proportion of participants from the same family was modest and balanced between groups. Given that the primary outcome was a biochemical measure and randomization was balanced across groups, analyses were conducted at the individual level.

Due to violations of normality of secondary outcomes, between-group differences in change from baseline were analyzed using ANCOVA with change score as the dependent variable. Furthermore, ANCOVA analyses, controlling for age, BMI, dietary vitamin D intake, physical activity (sedentary minutes), season, and skin pigmentation type, were conducted to assess the effect of intervention on change in grip strength, sensorimotor function, and cognitive function. In subgroup analyses, the ANCOVA was repeated to assess the effect of intervention during the extended winter period. Model assumptions were checked, including normality of residuals, homogeneity of regression slopes, and Levene’s test of equality. Statistical significance was set at *P* < 0.05, and 95% confidence intervals were reported. Subgroup analysis of change (post- minus preintervention) in immune markers and bone turnover markers in insufficient and sufficient participants who were age, sex, and BMI matched was conducted as exploratory analyses, not powered to detect significant differences. To correct for multiple comparisons, a Benjamini–Hochberg false discovery rate procedure was applied, adjusting the reported *P* values accordingly to control for type I errors and reduce the risk of false positives.

## Results

**FIGURE 1:**
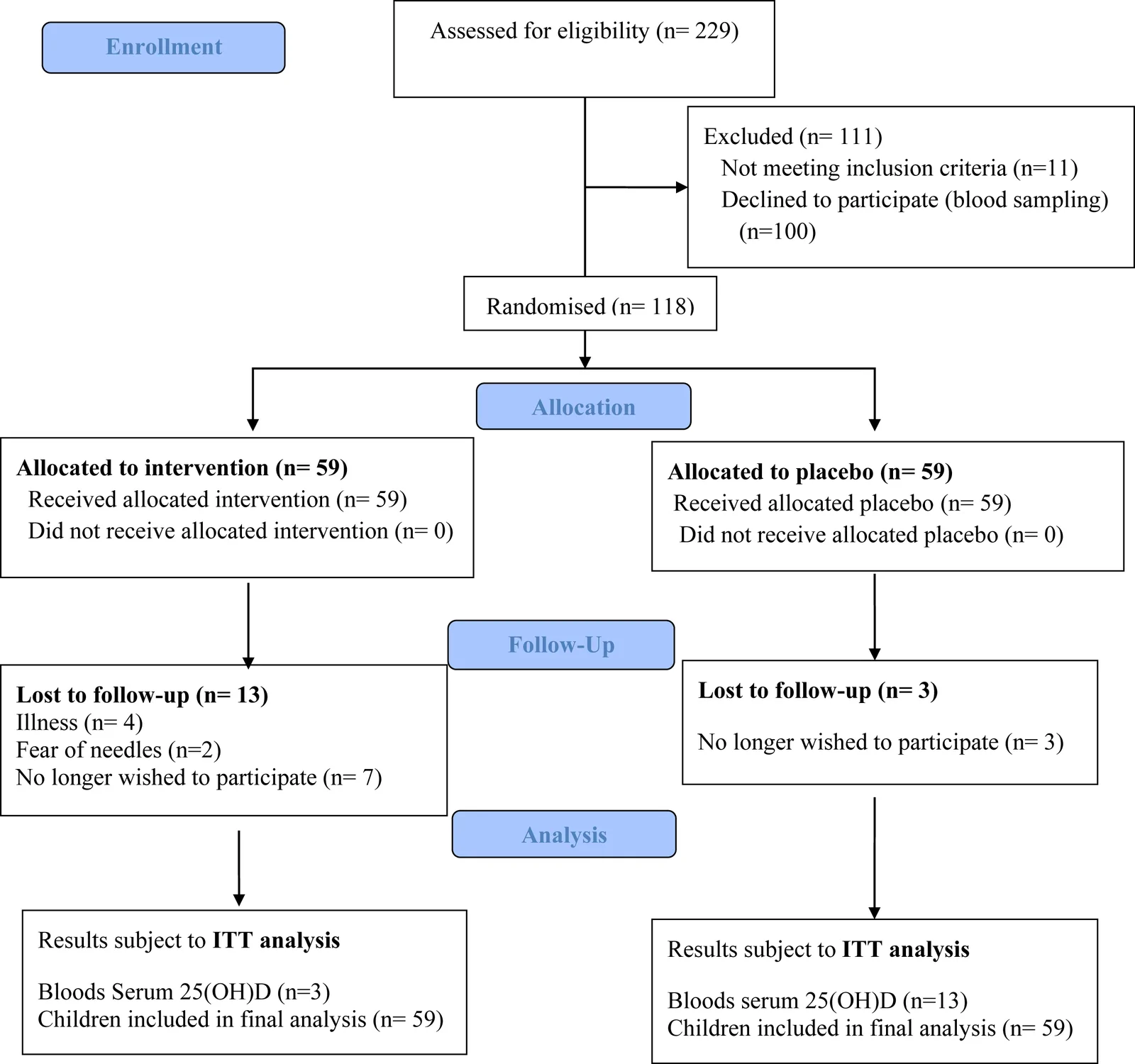
CONSORT flow diagram. A total of 229 families were assessed for eligibility with 111 excluded due to not meeting eligibility criteria and not wishing to participate. Remaining children were randomly assigned to receive either a 10 μg/d oral spray or matched placebo. CONSORT, Consolidated Standards of Reporting Trials.

Children’s baseline characteristics are outlined in Table 1. A total of 229 child families were screened, from which 118 children were recruited and randomly assigned to the intervention. In total, 77 families participated in the trial. Inclusion of siblings was permitted to facilitate recruitment; distribution of siblings was similar between groups, with 35 siblings in the placebo group and 37 siblings in the vitamin D group. A total of 102 children completed the intervention study in full, representing an 86% completion rate. A total of 16 children did not complete the study (intervention *n* = 13, placebo *n* = 3). In the vitamin D group, reasons for withdrawal included fear of needles/refusal of second blood sample (*n* = 3), illness (*n* = 4 from 1 family), parent forgot to give the spray and lost interest (*n* = 2 from 1 family), or were lost to follow-up (*n* = 4). In the placebo group, all 3 children withdrew without providing a reason (Figure 1). Although a larger number of participants withdrew from the vitamin D group compared with the placebo, withdrawals were due to a variety of reasons unrelated to the intervention, and baseline characteristics of those lost to follow-up were similar to those of participants who completed the study. Of the 118 children at baseline, 30 (25%) had a serum 25(OH)D concentration <50 nmol/L, indicative of insufficient status.

**TABLE 1:** Characteristics of D vitamin in children study participants at baseline (*n* = 118)

Treatment group	Total (*n* = 118)	Placebo (*n* = 59)	Vitamin D (*n* = 59)	*P*
variable		Baseline	Baseline	
Age (y)	8.2 ± 1.8	8.4 ± 1.9	8.3 ± 1.9	0.572
Sex (%)				0.581^1^
Male	58 (49.2)	31 (52.5)	27 (45.8)	
Female	60 (50.8)	28 (47.5)	32 (54.2)	
Height (cm)	133.3 ± 12.7	135.9 ± 11.6	134.3 ± 11.6	0.312
Weight (kg)	31.8 ± 9.2	33.1 ± 9.9	31.3 ± 8.5	0.339
BMI (kg/m^2^)	16.8 ± 2.19	17.5 ± 2.9	17.0 ± 2.2	0.570
BMI-for-age *z*-scores (kg/m^2^)^2^	0.47 ± 1.04	0.49 ± 1.04	0.45 ± 1.05	0.819
BMI Categories (%)				<0.001^1^
Thinness	2 (1.7)	0 (0)	2 (3.4)	
Normal	82 (69.5)	40 (67.8)	42 (71.2)	
Risk of overweight	24 (20.3)	15 (25.4)	9 (15.3)	
Risk of obesity	10 (8.5)	4 (6.8)	6 (10.2)	
Biomarkers				
25(OH)D concentration (nmol/L)	64.99 ± 18.37	63.67 ± 19.48	66.31 ± 17.25	0.436
25(OH)D concentration (min-max) (nmol/L)	(25.16–114.01)	(25.16–106.53)	(29.95–114.01)	
Vitamin D status (%)				0.056^1^
Insufficient	30 (25.4)	20 (33.9)	10 (17.0)	
Sufficient	88 (74.6)	39 (66.1)	49 (83.0)	
PTH (ng/L)	31.31 ± 13.28	32.95 ± 15.22	32.95 ± 15.22	0.176
Season (%)				0.937^1^
Spring	16 (13.6)	(15.3)	7 (11.9)	
Summer	19 (16.1)	(16.9)	9 (15.3)	
Autumn	37 (31.4)	18 (30.5)	19 (32.2)	
Winter	46 (39.0)	22 (37.3)	24 (40.7)	
Dietary vitamin D (*μ*g/d)	3.70 (2.65, 5.20)	3.34 (2.25, 4.80)	4.13 (2.80, 6.10)	0.358
Parent Education^3^ (%)				0.110^3^
Secondary education	11 (9.3)	1 (1.7)	10 (16.9)	
FE College	12 (10.1)	8 (13.6)	4 (6.8)	
Undergraduate (BSc.)	29 (24.6)	15 (25.4)	14 (23.7)	
Postgraduate (MSc., PhD.)	52 (44.1)	29 (49.1)	23 (39.0)	
Other	2 (1.7)	2 (3.4)	0 (0)	

All anthropometry measured in triplicate, mean obtained. All values are mean ± SD.

Abbreviations: PTH, parathyroid hormone; SD, standard deviation; WHO, World Health Organization; 25(OH)D, 25-hydroxyvitamin D.

1 χ^2^ test for categorical variables. *P* values are significant at *P* < 0.05.

2 Calculated using WHO AnthroPlus software.

3 Parent education data available for *n* = 106. Vitamin D status total population: insufficient = 30; sufficient = 88; placebo group: insufficient = 20; sufficient = 39; vitamin D group insufficient = 10; sufficient = 49. Vitamin D intakes from food sources only.

### Effect of vitamin D supplementation on plasma 25(OH)D

The effect of intervention on plasma 25(OH)D concentrations at week 12, adjusted for baseline 25(OH)D, age, BMI, dietary intake, season, skin pigmentation score, and physical activity (sedentary minutes), is shown in Table 2. Supplementation with 10 *μ*g/d significantly increased concentrations of 25(OH)D compared to no supplementation (*P* < 0.001). Postintervention, none of the supplemented children were deficient, and only 6 (10%) remained insufficient. In contrast, in the placebo group, 34% of the children were vitamin D insufficient, including 2 (∼4%) who were vitamin D deficient. Overall, there were no adverse effects of supplementation, and the oral spray was well tolerated.

**TABLE 2:** The effect of vitamin D supplementation on postintervention biomarker status

Biomarker	Placebo (n = 59)		Vitamin D (n = 59)		Between-group differences (95% CI)	P
	Baseline	Week 12	Baseline	Week 12		
Total 25(OH)D (nmol/L)	63.67 ± 2.75	57.67 ± 1.97	66.31 ± 2.44	69.35 ± 2.02	11.72 (5.96, 17.75)	<0.001
PTH (ng/L)	32.95 ± 15.22	35.81 ± 15.58	29.60 ± 10.73	30.24 ± 14.26	‒2.46 (‒7.75, 2.82)	0.357

Extended winter	Placebo (n = 34)		Vitamin D (n = 35)			

Total 25(OH)D (nmol/L)	55.15 ± 14.66	49.49 ± 14.19	61.07 ± 15.08	66.16 ± 16.60	15.81 (6.94, 24.68)	<0.001
PTH (ng/L)	36.18 ± 18.30	39.50 ± 16.34	30.92 ± 9.83	32.26 ± 14.28	‒1.98 (‒9.9, 5.9)	0.618

Baseline and week 12 data are presented descriptively; all inferential statistics are based on adjusted between-group comparisons. Results are presented as adjusted estimated marginal means with 95% CIs. Between-group differences were assessed using ANCOVA with postintervention as the dependent variable, adjusting for baseline 25(OH)D, BMI, dietary intake, age, physical activity (sedentary minutes), season, and skin pigment type. P values represent adjusted between-group treatment effects. Subgroup analyses were analyzed using the same ANCOVA framework. Between-group differences were assessed using ANCOVA with postintervention as the dependent variable, adjusting for baseline PTH, BMI, dietary intake, age, season, and skin pigment type. P values represent adjusted between-group treatment effects. Subgroup analyses were analyzed using the same ANCOVA framework. Missing data for 25(OH)D and PTH at week 12: last observation carried forward n = 16 (13.6%) for the total study population. Equality of variances across groups was verified using Levene’s test (P > 0.05), supporting the assumption of homogeneity of variances.

Abbreviations: ANCOVA, analysis of covariance; CI, confidence interval; PTH, parathyroid hormone; 25(OH)D, 25-hydroxyvitamin D.

### Effect of vitamin D supplementation on plasma 25(OH)D during the extended winter

Secondary analyses showed that 10 *μ*g/d significantly increased concentrations of 25(OH)D compared to no supplementation (*P* < 0.05) during the extended winter (November to March) (Table 2). Throughout this period of time, none of the supplemented children were vitamin D deficient, and ∼11% remained insufficient. In contrast, in the placebo group, 50% were insufficient, of which 1 child (∼3%) was vitamin D deficient (<25 nmol/L).

### Effect of vitamin D supplementation on secondary outcomes

iPTH concentrations were within normal reference ranges at baseline and follow-up in both groups. There were no significant changes in iPTH between groups (Table 2). There were no significant changes in absolute or relative grip strength between groups (Supplementary Table 1). There were no significant changes in time between groups in single-leg balance with eyes open or eyes closed, nor when balancing in tandem stance (Supplementary Table 2). There were no significant changes in measures of cognitive function (SSP, RTI movement time, RTI reaction time, RVP) between groups (Supplementary Table 3).

In exploratory subgroup analyses, there was no significant change in any immune markers regardless of vitamin D status at baseline (Supplementary Table 4). There was no significant change in the bone turnover markers CTX or P1NP postintervention regardless of baseline vitamin D status (Supplementary Table 5).

## Discussion

The findings from this study demonstrate for the first time in Northern Ireland that supplementing with 10 *μ*g/d vitamin D_3_ for 12 wk leads to a mean increase in circulating 25(OH)D concentrations and, in the majority, prevents vitamin D insufficiency in children aged 4‒11 y residing at a northerly latitude. During the extended winter, when the contribution of UVB exposure to vitamin D status is negligible, participants in the vitamin D group maintained or improved vitamin D status, bar 1 participant who became insufficient, whereas within the placebo group, 15% became insufficient. Beyond effects on vitamin D status, vitamin D_3_ supplementation was associated with nominal improvements in measures of psychomotor speed; however, these effects did not remain statistically significant following correction for multiple comparisons. No effects, however, were observed on grip strength or sensorimotor function. These findings highlight that although modest supplementation is generally effective for maintaining sufficiency and supporting neurocognitive outcomes, it may be inadequate to fully correct low baseline status or impact muscle and sensorimotor function in generally healthy children.

At baseline, the 25(OH)D concentrations were above the sufficiency threshold (>50 nmol/L); however, a quarter of children were vitamin D insufficient. Over the 12-wk intervention, on mean, 25(OH)D concentrations increased in the supplementation group but declined in the placebo group, irrespective of season. Notably, without supplementation, insufficiency worsened over 12 wk, with 34% in the placebo group vitamin D insufficient at endpoint, including 2 children who fell below the cut-off for vitamin D deficiency. In contrast, following supplementation, in those children who were insufficient at baseline (*n* = 10), vitamin D supplementation corrected insufficient status in 6 children. Nevertheless, ∼40% receiving supplementation also experienced decreases in 25(OH)D concentrations; however, these changes rarely resulted in a shift to insufficient status, with only 6 children dropping below sufficiency thresholds.

Beyond baseline status and ethnicity, the exact mechanism driving the variability in response to vitamin D supplementation has not yet been determined; however, other biological factors, including genetic and metabolic differences [33], are known to contribute. Variants in the vitamin D binding protein genotype have been associated with vitamin D deficiency in adolescents [34] and with an improved response to supplementation in infants [35]. Several vitamin D-related genes contribute to individual differences in supplementation response in children. Single-nucleotide polymorphisms in key genes, including the vitamin D receptor (VDR), vitamin D-binding protein (GC gene), cytochrome P450 2R1 (CYP2R1), and retinoid X receptor alpha (RXRA), have been associated with altered vitamin D metabolism, transport, and receptor activity [36]. These genetic factors, when considered alongside environmental [37] and societal factors [38], may help explain why some supplemented children did not increase their 25(OH)D concentrations. These findings suggest that although 10 *μ*g/d is broadly effective at maintaining vitamin D sufficiency for most children, it may be inadequate to fully correct low status in all children.

Seasonal effects were most evident during the extended winter (November to March), when the contribution of UVB exposure to vitamin D status is negligible, and reliance upon dietary intake and vitamin D stores is essential. Among the 69 children enrolled during this period, baseline 25(OH)D concentrations did not differ between the supplementation and placebo groups. Following intervention, however, children who received supplementation achieved a sufficient status on mean, whereas those not supplemented experienced a mean decline in 25(OH)D concentrations, resulting in a mean insufficient status. Although this subgroup was not powered to detect significant differences, the results highlight the seasonal vulnerability of children’s vitamin D status and reinforce the importance of consistent strategies to maintain sufficient status. Comparable intervention studies in European children have also reported mean increases in 25(OH)D concentrations of 5–25 nmol/L following supplementation with ≤20 *μ*g/d [20,21], reinforcing that inter-individuality is common, and higher doses may be required to optimize status across the population.

Generalizability of these findings to the broader Northern Ireland population should be considered in the context of the socioeconomic and ethnic profile of Northern Ireland. Recent Northern Ireland census results indicate that ∼12% of individuals live in relative poverty, with child poverty rates higher than the population mean [39]. The region is also relatively ethnically homogeneous compared with other parts of the UK, with ∼96.6% of residents identifying as Caucasian and 3.4% belonging to minority groups [40]. Consistent with this population profile, the study sample was predominantly Caucasian and skewed toward more socioeconomically advantaged families. Although this reflects the local demographic context, it may limit generalizability to more ethnically diverse or socioeconomically disadvantaged populations, in whom vitamin D status, sun exposure behaviors, and dietary patterns may differ, and further research in these populations is warranted.

Although no cognitive outcomes remained statistically significant following adjustment for multiple testing, observed trends are biologically plausible given the known role of vitamin D in brain development and function. Vitamin D supplementation potentially stimulated improved motor execution speed; in contrast, the placebo group showed a decline; this divergent trajectory suggests a protective and enhancing role of vitamin D on neuromotor function in children. To our knowledge, this is the first time this has been shown in children as assessed using the well-validated Cambridge Neuropsychological Test Automated Battery (CANTAB). Micronutrient deficiencies are linked to brain health and cognitive function [41]. Vitamin D is thought to exert its effects through broad expression of vitamin D receptors and metabolites in brain regions related to learning, memory, and motor control [[42], [43], [44]]. Hydroxylating enzymes in neurons and glia cells further enable metabolism, supporting a regulatory role in neuroplasticity within motor-related brain domains such as the cerebellum and basal ganglia [45]. Furthermore, these findings are consistent with previous research conducted in adolescents aged 12‒18 y, suggesting improved attention, memory, and inhibitory control following supplementation [46] and improved gross motor function in infants aged 3‒6 mo following modest supplementation [47]. These findings suggest that cognitive and psychomotor domains may be particularly sensitive to improvements in vitamin D status, even over relatively short intervention periods. The observed improvement may therefore reflect the role of vitamin D in neurodevelopmental processes, particularly during ongoing maturation of the prefrontal cortex during late childhood [48,49].

Vitamin D supplementation had no significant effect on grip strength and balance, which remained following correction for multiple comparisons. This may reflect the relatively short intervention duration and the differing physiological mechanisms underlying these outcomes [50]. Grip strength is a proxy for muscle hypertrophy and contractile force, which may require longer supplementation periods or higher doses to demonstrate measurable improvements [51]. Furthermore, grip strength and balance measures are subject to developmental and measurement variability in child populations, which makes small changes harder to detect [52]. A previous study reporting improvements in muscle performance included jumping mechanography and higher doses provided to a more deficient population, suggesting sensorimotor outcomes may require greater stimulus to show measurable change [6]. Our findings, therefore, align with the idea that vitamin D status may positively influence cognitive and psychomotor functions before measurable changes in gross motor function are observed in generally healthy children.

Dietary intake was limited, with children obtaining only ∼30% of the recommended vitamin D from diet. Only 3 children met the national guidelines (10 *μ*g/d), and fortified (voluntary in the UK) cereals were the greatest contributor to their vitamin D intakes. It is worth noting that all 3 children were in the vitamin D supplement group. Notably similar low intakes have been reported in other child cohorts [16,20,21,53,54], reflecting the limited contribution of vitamin D-rich foods to children’s diets. Supplementation with 10 *μ*g/d, although below the recommended dietary allowance of 15 *μ*g/d, helps bring total vitamin D intake (from diet and supplements) closer to the typical 15 *μ*g/d recommended dietary allowance for this age group. These findings reinforce the need for supplementation in this age group in the absence of (bio)fortification policies [55].

In the subgroup that was vitamin D sufficient at baseline, nominal differences were observed between groups, with those receiving supplements having higher IL-4 and TNF-α when compared to placebo. Notably, in the vitamin D sufficient group, supplementation raised mean 25(OH)D concentrations from ∼74 nmol/L to ∼82 nmol/L, whereas the placebo group decreased from ∼72 nmol/L to ∼58 nmol/L. Although these differences did not remain significant following correction for multiple comparisons and should be cautiously interpreted. Higher IL-4 and TNF-α in those with better vitamin D status may be indicative of their ability to maintain a more balanced T helper 1/T helper 2 cell (Th1/Th2) profile. Thus, suggesting that maintaining 25(OH)D >75 nmol/L may contribute to immune function homeostasis and prevent fluctuations that occur with marginal decline in vitamin D status [56]. Evidence for vitamin D’s effect on inflammatory markers in children remains mixed, with some studies reporting no effects on IL-6 or IL-4 [56,57] but overall supporting a role for vitamin D in modulating immune responses.

In subset analysis, vitamin D supplementation had no significant effect, compared to placebo, on the bone turnover markers CTX and P1NP, irrespective of baseline vitamin D status, indicating no beneficial effect of supplementation on bone metabolism. However, the interpretation of these results is constrained by the limited sample size. Similarly, a previous meta-analysis found no effect of vitamin D supplementation on bone markers in children albeit the data were too limited to determine if bone markers are a robust vitamin D biomarker in children [58]. Nonetheless, these data are consistent with findings from a recent systematic review and individual participant data meta-analysis of randomized controlled trials, which collectively indicate that vitamin D supplementation is not warranted for improving bone health in children in the absence of severe vitamin D deficiency [59].

Alongside bone turnover markers, nonfasting plasma iPTH was assessed to provide additional insight into calcium-vitamin D homeostasis. In the overall study population, a modest increase in iPTH was observed in both the vitamin D and placebo groups, albeit nonsignificant, which was also observed among participants recruited throughout the winter months. Given that baseline iPTH was largely within normal reference range, and iPTH was not a primary outcome, these results should be interpreted cautiously. The observed seasonal variation may reflect the influence of limited UVB exposure and other physiological factors on parathyroid hormone regulation rather than a direct effect of supplementation alone.

### Strengths and limitations

Strengths and limitations of the current study are noteworthy. To our knowledge, this is the first randomized controlled trial that examined the effects of 10 *μ*g/d vitamin D supplementation in children with study enrolment throughout the year within the province of Ulster. The current results were observed in children who were, on mean, vitamin D sufficient at baseline, which may have influenced the response to supplementation. Future research is required to confirm if this dose/duration of supplementation is adequate for those with lower vitamin D status at baseline. Children from the same family were included in the trial, with siblings assigned to the same treatment group to avoid within-household cross-contamination and reflect real-world supplementation practices. Analyses were conducted at the individual level, and family-level factors, including shared environment and individual behaviors, may have influenced outcomes. The randomized controlled trial design and high compliance (>85%) support the internal validity of the findings. The use of an oral spray formulation, previously shown to be as effective as capsules [60], also represents a practical and well-tolerated method of supplementation that may be particularly attractive for younger children who have difficulty taking capsules/tablets. The vitamin D_3_ spray was a commercially manufactured product with a labeled dose of 10 *μ*g per spray. Products were supplied in sealed containers, stored according to manufacturer recommendations, and used within their stated expiry date. Although no formal laboratory verification of overage was conducted, use of a commercially available product reflects real-world supplementation practices, which is important in this study. Recruitment throughout the year allowed seasonal patterns in vitamin D status to be observed, enhancing the generalizability of the results to populations at a northern latitude. Furthermore, although the current study was only powered to detect changes in vitamin D status as the primary outcome, the simultaneous assessment of multiple secondary outcomes, although exploratory in nature, provides an integrative view of vitamin D’s effects during childhood. Data reported here on health outcomes will help to inform future research in this area, in which studies may need to be longer in duration to detect changes in outcomes that adapt more slowly, such as grip strength or bone metabolism. Moreover, future research investigating the effect of vitamin D on functional measurements should consider a smaller age range of children to minimize developmental variability. Future research could consider screening at baseline for vitamin D deficiency and/or insufficiency to inform on the effects of supplementation in those children at risk of deficiency. Importantly, it should also be noted that this study was carried out pre and post the COVID-19 pandemic lockdown, where media attention was focused upon vitamin D and may have influenced health knowledge and behaviors.

In conclusion, current public health strategies for vitamin D supplementation are insufficient to ensure adequacy in all children. Our findings show that 10 *μ*g/d vitamin D_3_ maintains vitamin D sufficiency for most, but some children remained insufficient, particularly during winter. Supplementation was effective at maintaining vitamin D sufficiency, whereas small improvements in attention and psychomotor speed were observed, highlighting potential broader neurodevelopmental benefits. Diet alone is inadequate, with most children only achieving a fraction of recommended levels. These results highlight the urgent need for targeted research in a population with insufficiency at baseline, over the extended winter months, to determine solely the effect of supplementation on nonskeletal health outcomes. More broadly, these findings call for consideration of higher or tailored doses for at-risk children in the absence of mandatory fortification to safeguard vitamin D status across child populations.

## Author contributions

The authors’ responsibilities were as follows – EMM, LKP, DJA, RRI, JTM, NG, PJM: designed the research; JEM, LG, COH, MMS, DUG, ER: conducted the research; ER: analyzed data; ER, EMM, LKP, PJM: wrote the article; PJM: had primary responsibility for final content; and all authors: read and approved the final manuscript.

## Data availability

Data described in the manuscript, code book, and analytic code will be made available from the corresponding author upon request, pending application and approval.

## Declaration of Generative AI and AI-assisted technologies in the writing process

The authors declare that no generative AI or AI-assisted technologies were used in the writing of this manuscript.

## Funding

This study was supported by grants from the Department for the Economy (DfE), a Queen Margaret University PhD studentship, and BetterYou Ltd to support the analysis of immune and bone turnover markers. Additionally, BetterYou Ltd provided the supplement and placebo control sprays for the study. This project was completed in collaboration with Queen Margaret University. Neither DfE nor BetterYou Ltd had any involvement in the study design, data collection, analysis, or interpretation.

## Conflict of interest

The authors report no conflicts of interest.
