# Supplementary material for: The Effect of Vitamin D3 Supplementation on Vitamin D Status and Associated Health Outcomes in Children: A Randomized Controlled Trial
Source: J Nutr. 2026 Mar 24;156(8):101493. doi: 10.1016/j.tjnut.2026.101493 (PMC13494196; doi:10.1016/j.tjnut.2026.101493)
Supplement: Supplementary file 1 [file mmc1.docx]

**Supplementary Table 1.** The effect of vitamin D supplementation on grip strength (change from baseline).

| **Grip strength** | **Placebo (n=59)** | **Vitamin D (n=59)** | **Adjusted between-group difference in change (95% CI)** | ***P (FDR)*** |
| --- | --- | --- | --- | --- |
|  | ***Mean change (adjusted)± SE*** | ***Mean change (Adjusted)± SE*** |  |  |
| *Absolute grip strength* |  |  |  |  |
| Average Peak Strength | 0.41 ± 0.25 | 0.387 ± 0.25 | -0.02 (-0.74, 0.69) | 0.99 |
| Maximum Peak Strength | 0.60 ± 0.29 | 0.564 ± 0.29 | -0.03 (-0.87, 0.80) | 1.00 |
| Maximum Dominant Peak Strength | 0.37 ± 0.30 | 0.492 ± 0.31 | 0.12 (-0.74, 0.99) | 1.00 |
| *Relative grip strength* |  |  |  |  |
| Average Peak Strength | 0.03 ± 0.02 | 0.03 ± 0.02 | -0.01 (-0.43, 0.42) | 1.00 |
| Maximum Peak Strength | 0.04 ± 0.02 | 0.04 ± 0.02 | 0.00 (-0.05, 0.05) | 1.00 |
| Maximum Dominant Peak Strength | 0.02 ±0.02 | 0.03 ± 0.02 | -0.01 (-0.60, 0.39) | 1.00 |

Abbreviations: SE, Standard error of the mean; CI, Confidence intervals; FDR, Benjamini-Hochberg false discovery rate procedure; All inferential statistics are based on adjusted between-group comparisons, presented as adjusted estimated marginal means with 95% confidence intervals. Between group differences were assessed using ANCOVA with change in the dependent variable, adjusting for baseline grip strength, BMI, dietary intake, age, physical activity (sedentary minutes), season, and skin pigment type. P-values adjusted for multiple comparisons using the Benjamini-Hochberg false discovery rate procedure (P-FDR). No missing data for grip strength at week 12. Equality of variances across groups were verified using Levene’s test (p>0.05), supporting the assumption of homogeneity of variances.

**Supplementary Table 2.** The effect of vitamin D supplementation on sensorimotor function (change from baseline).

| **Sensorimotor outcomes** | **Placebo (n=59)** | **Vitamin D (n=59)** | **Adjusted between-group difference in change (95% CI)** | ***P (FDR)*** |
| --- | --- | --- | --- | --- |
|  | ***Mean change (Adjusted)* *± SE*** | ***Mean change (Adjusted*) *± SE*** |  |  |
| *Single leg stance balance (seconds)* |  |  |  |  |
| Eyes open | 2.65 ± 1.90 | 2.52 ± 1.96 | -0.13 (-5.42, 5.67) | 1.00 |
| Eyes closed | 0.82 ± 1.80 | 1.31 ± 1.82 | -0.50 ( -5.65, 4.66) | 1.00 |
| *Tandem leg stance balance (seconds)* |  |  |  |  |
| Eyes open | 3.04 ± 2.19 | 2.35 ± 2.26 | 0.69 (-5.74, 7.11) | 1.00 |
| Eyes closed | 3.23 ± 2.49 | 1.83 ± 2.62 | 1.40 (-6.06, 8.86) | 1.00 |

Abbreviations: SE, Standard error of the mean; CI, Confidence intervals; FDR, Benjamini-Hochberg false discovery rate procedure; All inferential statistics are based on adjusted between-group comparisons, presented as adjusted estimated marginal means with 95% confidence intervals. Abbreviations: SE, standard error Between group differences were assessed using ANCOVA with change in the dependent variable, adjusting for baseline balance, BMI, dietary intake, age, physical activity (sedentary minutes), season, and skin pigment type. P-values adjusted for multiple comparisons using the Benjamini-Hochberg false discovery rate procedure (P-FDR). Missing data for single leg stance eyes closed (n=4), Tandem leg stance Eyes closed (n=5), Tandem leg stance Eyes open (n=3). Last observation carried forward at week 12. Equality of variances across groups were verified using Levene’s test (p>0.05), supporting the assumption of homogeneity of variances.

**Supplementary Table 3**. The effect of vitamin D supplementation on cognitive function (change from baseline).

| **Cognitive function tests** | **Placebo (n=50)** | **Vitamin D (n=55)** | **Adjusted between-group difference in change (95% CI)** | | | ***P (FDR)*** |  |
| --- | --- | --- | --- | --- | --- | --- | --- |
|  | ***Mean change (Adjusted)± SE*** | ***Mean change (Adjusted) ± SE*** | |  |  | | |
| *Attention* |  |  | |  |  | | |
| SSP (span length) | 4.4 ± 0.2 | 4.4 ± 0.2 | | 0.3 (-0.5, 0.6) | 0.95 | | |
| *Movement Time (milliseconds)* |  |  | |  |  | | |
| RTI Simple | 7.7 ± 46.3 | -109.4 ± 48.7 | | -117.0 (-255.9, 21.9) | 0.95 | | |
| RTI 5 Choice | -6.4 ± 21.0 | -61.6 ± 22.1 | | 55.2 (-118.4, 7.9) | 0.95 | | |
| *Rection Time (Milliseconds)* |  |  | |  |  | | |
| RTI Simple | -28.3 ± 13.9 | -6.6 ± 14.6 | | 21.7 (-20.0, 63.5) | 0.95 | | |
| RTI 5 Choice | 1.8 ± 12.6 | 14.3 ± 13.3 | | 12.6 (-25.4, 50.5) | 0.95 | | |
| RVP Latency | -65.3 ± 10.1 | -48.6 ± 11.1 | | 16.7 (-14.2, 47.6) | 0.95 | | |

Abbreviations: SE, Standard error of the mean; CI, Confidence intervals; FDR, Benjamini-Hochberg false discovery rate procedure; SSP, Spatial span; RTI, reaction time; RVP, rapid visual processing; All inferential statistics are based on adjusted between-group comparisons, presented as adjusted estimated marginal means with 95% confidence intervals. RTI, five-choice reaction time measured as reaction time latency (milliseconds); SSP, spatial span measured as longest sequence length recalled correctly, RTI simple and five-choice movement time measured as mean movement time (milliseconds). Between group differences were assessed using ANCOVA with change in the dependent variable, adjusting for baseline cognitive test, BMI, dietary intake, age, season, and skin pigment type. P-values adjusted for multiple comparisons using the Benjamini-Hochberg false discovery rate procedure (P-FDR). Missing data for SSP (n=10), RTI simple movement time (n=8), RTI 5 choice movement time (n=9), RTI simple reaction time (n=8), RTI 5 choice reaction time (n=9), RVP Latency (n=9). Nine children did not complete testing due to tiredness. Last observation carried forward at week 12. Equality of variances across groups were verified using Levene’s test (p>0.05), supporting the assumption of homogeneity of variances

**Supplementary Table 4.** The effect of vitamin D supplementation on immune markers (change from baseline) stratified by vitamin D status at baseline

| **Cytokines** | ***Mean change (Adjusted)***  ***± SE*** | ***Mean change (Adjusted)***  ***± SE*** | ***Adjusted between-group difference in change (95% CI)*** | ***P (FDR)*** |
| --- | --- | --- | --- | --- |
| *Sufficient 25(OH)D (≥50 nmol/L)* | ***Placebo (n=20)*** | ***Vitamin D (n=7)*** |  |  |
| IL-1β | -14.80 ± 22.84 | 89.97 ± 49.52 | 104.77 (-23.77, 233.31) | 0.49 |
| IL-6 | 971.49 ± 757.67 | 305.86 ± 1637.66 | -665.63 (-4197.07, 2557.30) | 0.49 |
| IL-8 | -6.55 ± 14.45 | 55.92 ± 31.85 | 62.48 (-20.56, 145.51) | 0.49 |
| TNF-α | -380.54 ± 72.86 | 58.39 ± 166.79 | 438.94 (-0.56, 877.93) | 0.49 |
| IFN-γ | -251.51 ± 310.85 | -782.86 ± 662.76 | -531.36 (-2243.95, 1181.24) | 0.49 |
| IL-2 | 12.99 ± 17.01 | -25.77 ± 36.39 | -38.75 (-132.73, 55.21) | 0.49 |
| IL-4 | -36.59 ± 18.08 | 7.04 ± 42.59 | 43.63 (-69.20, 156.46) | 0.49 |
| IL-10 | -356.82 ± 208.16 | -303.85 ± 471.98 | 52.98 (-1186.37, 1292.33) | 0.49 |
| IL12p70 | -94.38 ± 54.67 | -82.31 ± 115.48 | 12.07 (-285.54, 309.68) | 0.49 |
| IL-13 | -18.64 ± 14.31 | -11.73 ± 30.15 | 6.91 (-70.73, 84.54) | 0.49 |
| CRP ^++^ | -0.1209 ± 0.2132 | 0.407 ± 0.406 | 0.503 (-0.605, 0.174) | 0.49 |
| *Insufficient 25(OH)D (≤50 nmol/L)* | ***Placebo (n=19)*** | ***Vitamin D (n=8)*** |  |  |
| IL-1β | 22.48 ± 22.92 | 34.22 ± 33.82 | 11.75 (-75.69, 99.18) | 0.54 |
| IL-6 | 1053.46 ± 885.53 | 233.57 ± 1306.36 | -819.89 (-4197.07, 2557.30) | 0.85 |
| IL-8 | 2.31 ± 5.76 | -1.81 ± 8.53 | -4.12 (-26.22, 17.98) | 1.00 |
| TNF-α | -131.48 ± 59.94 | -56.84 ± 89.97 | 74.64 (-161.29, 310.57) | 1.00 |
| IFN-γ | 253.78 ± 182.78 | 265.66 ± 269.56 | 11.88 (-685.28, 709.03) | 0.54 |
| IL-2 | 49.97 ± 36.74 | 53.76 ± 55.92 | 3.80 (-144.43, 152.02) | 0.92 |
| IL-4 | -42.30 ± 15.79 | -24.19 ± 23.34 | 18.11 (-42.33, 78.55) | 0.62 |
| IL-10 | 168.37 ± 261.20 | 45.37 ± 386.99 | -123.03 (-1127.12, 881.05) | 0.52 |
| IL12p70 | -31.68 ± 44.79 | -8.75 ± 66.07 | 22.93 (-147.84, 193.70) | 0.54 |
| IL-13 | -12.70 ± 24.71 | 37.96 ± 36.51 | 50.66 (-145.15, 43.84) | 0.52 |
| CRP ^++^ | 0.205 ± 0.108 | 0.207 ± 0.207 | 0.29 (-0.608, 0.704) | 0.72 |

Abbreviations: 25(OH)D, 25-hydroxyvitamin D; IL-1β, Interleukin – 1Beta; IL-6, Interleukin -6; IL-8, Interleukin- 8; TNF-α, Tumour necrosis factor- alpha; IFN-γ, Interferon - gamma; IL-2, Interleukin-2; IL-4, interleukin-4; IL-10, Interleukin-10; IL12p70, Interleukin-12p70; IL-13, Interleukin-13; CRP, C-reactive protein; SE, Standard error of the mean; CI, Confidence intervals; FDR, Benjamini-Hochberg false discovery rate procedure. All cytokine concentrations are in fg/ml unless ^++^ which denotes ng/mL and are given as mean change ± standard error. Between group differences were assessed using ANCOVA with change in the dependent variable, adjusting for baseline cognitive test, BMI, dietary intake, age, season, and skin pigment type. P-values adjusted for multiple comparisons using the Benjamini-Hochberg false discovery rate procedure (P-FDR). Equality of variances across groups were verified using Levene’s test (p>0.05), supporting the assumption of homogeneity of variances.

| **Bone turnover marker** | ***Mean change (Adjusted) ± SE*** | ***Mean change (Adjusted) ± SE*** | ***Adjusted between-group difference in change (95% CI)*** | ***P (FDR)*** |
| --- | --- | --- | --- | --- |
| *Sufficient 25(OH)D (>50 nmol/L)* | ***Placebo (n=20)*** | ***Vitamin D (n=7)*** |  |  |
| CTx | 0.086 ± 0.125 | 0.549 ± 0.412 | 0.462 (-0.751, 1.676) | 1.00 |
| PINP | 46.86 ± 43.52 | -95.25 ± 182.84 | -142.12 (-690.75, 460.52) | 1.00 |
| *Insufficient 25(OH)D (<50 nmol/L)* | ***Placebo (n=19)*** | ***Vitamin D (n=8)*** |  |  |
| CTx | -0.023 ± 0.178 | -0.056± 0.263 | -0.033 (-0.917, 0.851) | 1.00 |
| PINP | 18.29 ± 19.16 | 14.33 ± 39.91 | -3.96 (-149.81, 141.88) | 1.00 |

**Supplementary Table 5.** The effect of vitamin D supplementation bone turnover markers (change from baseline) stratified by vitamin D status at baseline.

Abbreviations: 25(OH)D, 25-hydroxyvitamin D; SE, Standard error of the mean; CI, Confidence intervals; FDR, Benjamini-Hochberg false discovery rate procedure; CTx, Serum cross-linked C-telopeptide, concentrations are in pg/ml; PINP, Procollagen type 1 N propeptide; concentrations are in μg/ml and are given as mean change ± Standard error. . Between group differences were assessed using ANCOVA with change in the dependent variable, adjusting for baseline cognitive test, BMI, dietary intake, age, season, and skin pigment type. P-values adjusted for multiple comparisons using the Benjamini-Hochberg false discovery rate procedure (P-FDR). Equality of variances across groups were verified using Levene’s test (p>0.05), supporting the assumption of homogeneity of variances.
